
JOURNAL INFORMATION
==============================
NLM Title Abbreviation: ScientificWorldJournal
Journal ID: ScientificWorldJournal
NLM Journal ID: 101131163
Journal ID: TSWJ

The Scientific World Journal

ISSN: 2356-6140
EISSN: 1537-744X
Publisher: Wiley

ARTICLE INFORMATION
==============================
PMCID: PMC6084096
PMID: 30147586
DOI: 10.1100/tsw.2001.120
Article ID: 467207
Article version: 1
Subjects: Short Report

Hyaluronic Acid and Hyaluronidase: Accurate Tumor Markers and Tumor Progression Regulators

Lokeshwar Vinata *
Schroeder Grethchen
Hautmann Stefan
Selzer Marie
Department of Urology, University of Miami School of Medicine, P.O. Box 016960, Miami, Florida 33101, USA
*Vinata Lokeshwar: vlokeshw@med.miami.edu
Publication date: 2001
Electronic publication date: 2001 Oct 23
Volume: 1
First page: 106
Last page: 106
Copyright: Copyright © 2001 Vinata Lokeshwar et al.
Copyright year: 2001
License: This is an open access article distributed under the Creative Commons Attribution License, which permits unrestricted use, distribution, and reproduction in any medium, provided the original work is properly cited.
License URL: https://creativecommons.org/licenses/by/3.0/

Funding: National Cancer Institute R29 CA 72821

==============================
Miami *Nature Biotechnology* Short Reports
*TheScientificWorld* (2001) 1(S3), 106SR
ISSN 1532-2246; DOI 10.1100/TSW.2001.120

### HYALURONIC ACID AND HYALURONIDASE: ACCURATE TUMOR MARKERS AND TUMOR PROGRESSION REGULATORS

Vinata Lokeshwar*, Grethchen Schroeder, Stefan Hautmann, and Marie Selzer

Department of Urology, University of Miami School of Medicine, P.O. Box 016960,
Miami, Florida 33101, USA,
*vlokeshw@med.miami.edu

**INTRODUCTION.** Identification of molecular determinants that regulate tumor progression would help in improving diagnosis and prognosis for cancer patients. Hyaluronic acid (HA) is a non-sulfated glycosaminoglycan that promotes tumor metastasis. Hyaluronidase (HAase) is an endoglycosidase that degrades HA into small angiogenic fragments. In an earlier study we demonstrated that measurement of urinary levels of HA and HAase (**HA-HAase test**) is > 90% accurate in detecting bladder cancer (BCa) and evaluating its grade. In this study, we examined the ability of the HA-HAase test to monitor patients for BCa recurrence and examined the effect of HA fragments, isolated from patient samples, on endothelial cell functions.

**METHODS.** The HA-HAase test was used to detect BCa recurrence. Cell proliferation, phosphotyrosine immunoblotting and biotinylated HA-binding protein blotting assays were used to study the effect of HA and HA fragments on endothelial cells. Tumor-associated HAase was identified by RT-PCR cloning and immunoblotting.

**RESULTS.** In a blinded study consisting of 223 urine specimens from 72 patients with a history of BCa, the HA-HAase test detected 161 out of 178 BCa cases (sensitivity of 90.4%). Out of the 45 clinically negative cases for BCa, the HA-HAase test was negative in 32 cases (71.7% specificity). Interestingly, 8 out of the 13 cases, which were false positive on the HA-HAase test, showed a true clinical recurrence within 3 to 9 months. Analysis of urinary HA profile using gel filtration chromatography showed the presence of angiogenic and high molecular mass HA species in the urine of BCa patients (Fig. 1). The HA and angiogenic HA fragments isolated from patient urine induced proliferation of human lung microvessel endothelial cells in a concentration-dependent manner. The angiogenic HA fragment F1, induced a 2.5-fold mitogenic response in human microvessel endothelial cells. In a biotinylated HA-blotting experiment, RHAMM was found to be the major HA receptor on endothelial cells. The HA and HA fragments, interacting through RHAMM induced protein tyrosine phosphorylation of p125FAK, paxillin and p42/p44 ERK in a dose and time dependent manner (Fig. 2). Using RT-PCR and immunoblot analysis, we established HYAL1 as the major HAase expressed in tumors (prostate and bladder). Immunohistochemical analysis demonstrated increased expression of HA in both tumor cells and tumor-associated stroma, whereas, HYAL1 was localized exclusively in tumor cells.

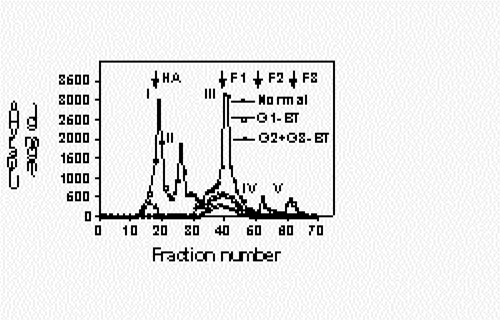

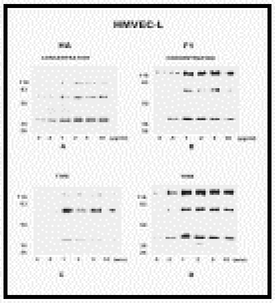

**DISCUSSION.** The results demonstrate that the HA-HAase test is an accurate test for detecting BCa occurrence and recurrence. The HA and HA fragments may induce angiogenesis by stimulating endothelial cell functions (e.g., adhesion, migration and proliferation) that involve the focal adhesion kinase and the MAP kinase pathways. The tissue expression of HA and HYAL1 may be important in predicting prognosis for BCa patients.

**ACKNOWLEDGEMENTS.** We thank Dr. Mark S. Soloway for providing clinical specimens for this study. This work was supported by NCI grant R29 CA 72821 and Sylvester Comprehensive Cancer Center, Univ. Miami.

**REFERENCES.**

1. Toole, B.P. (2000) J. Clin. Invest.106, 335-336
2. Lokeshwar, V.B., Obek, C., Pham, H.T., Wei, D.C., Young, M.J., Duncan, R.C., Soloway, M.S., and Block, N.L. (2000) J. Urol. 163, 348-356
3. Lokeshwar, V.B. and Selzer, M.G. (2000) J. Biol. Chem. 275, 27641-27649
